# Alpha-1 Antitrypsin Deficiency Presenting with MPO-ANCA Associated Vasculitis and Aortic Dissection

**DOI:** 10.1155/2017/8140641

**Published:** 2017-03-06

**Authors:** Bram M. Voorzaat, Jan van Schaik, Stijn L. P. Crobach, Catharina S. P. van Rijswijk, Joris I. Rotmans

**Affiliations:** ^1^Department of Nephrology, Leiden University Medical Center, Leiden, Netherlands; ^2^Department of Surgery, Leiden University Medical Center, Leiden, Netherlands; ^3^Department of Pathology, Leiden University Medical Center, Leiden, Netherlands; ^4^Department of Radiology, Leiden University Medical Center, Leiden, Netherlands

## Abstract

The combination of alpha-1 antitrypsin (AAT) deficiency, ANCA-vasculitis, and aortic aneurysm has been rarely described in literature. We report an eventually fatal case in a 70-year-old patient who initially presented with giant cell arteritis and ANCA associated glomerulonephritis. Several years later, he presented with aortic dissection due to large vessel vasculitis, raising the suspicion of AAT deficiency, as two first-line relatives had chronic obstructive pulmonary disease, while they never smoked. This diagnosis was confirmed by AAT electrophoresis and immunohistochemistry on a temporal artery biopsy. Considering AAT deficiency in these cases might lead to a more timely diagnosis.

## 1. Background

Alpha-1 antitrypsin (AAT) is part of the serine proteinase inhibitor family, primarily secreted by hepatocytes. By inhibiting proteinase activity, AAT protects against tissue damage [1]. Several mutations in the AAT-encoding gene SERPINA1 result in the polymerization of AAT in hepatocytes, thereby inhibiting its release into the circulation [2].

AAT deficiency is best known for pulmonary emphysema and liver cirrhosis. Other diseases including aortic aneurysm have been associated with AAT deficiency [3, 4]. Furthermore, AAT deficiency has been reported in cases of ANCA associated vasculitis (AAV). The strongest relationship has been described for antiproteinase (PR3) AAV, with a weaker association for antimyeloperoxidase (MPO) AAV [5].

Here, we present a patient with anti-MPO AAV and aortic dissection, later diagnosed with AAT deficiency.

## 2. Case Presentation

A 70-year-old man presented with fatigue, dyspnoea, and weight loss since two weeks. There were no cough and haemoptysis. He was not using any medication and has never smoked. The medical history was significant for temporal giant cell arteritis (GCA) two years earlier, which remained in remission after nine months of corticosteroids. Eight years earlier, spirometry was normal. His family history was significant for chronic obstructive pulmonary disease (COPD) in his father, sister, and brother, while his brother and sister never smoked.

Anti-MPO ANCA associated glomerulonephritis was diagnosed based on impaired renal function (eGFR CKD-EPI 14 mL/min/1.73 m^2^) with glomerular erythrocyturia, elevated inflammation parameters, and anti-MPO ANCA antibodies (15 U/L). The kidneys appeared normal on abdominal ultrasound, with an abdominal aorta of 2.6 cm. No kidney biopsy was performed. Cyclophosphamide 100 mg q.d. and prednisolone 60 mg q.d. were initiated. The symptoms remitted, the anti-MPO ANCA titre decreased to <1 IU/mL, and renal function improved to an eGFR of 40. The treatment was tapered and discontinued after one year.

Eighteen months after the diagnosis, he presented with severe back pain of sudden onset since two days. Blood pressure was 170/82 mmHg, peripheral pulsations were normal, and no bruits were heard.

## 3. Investigations

**Figure 1:**
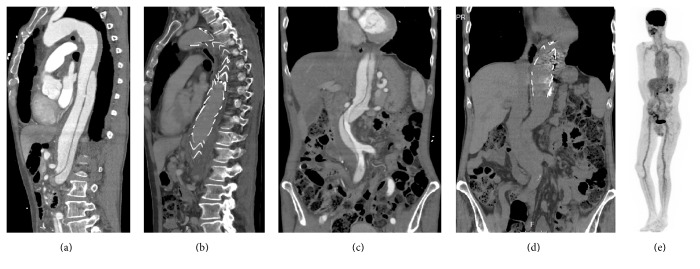
Radiological investigations. Sagittal ((a) and (b)) and coronal ((c) and (d)) reconstructions of CT scans, demonstrating type B aortic dissection and elongated aorta before and after TEVAR. The entry tear is demonstrated at the level of thoracic 10. 18-Fludeoxyglucose PET scan (e), demonstrating large vessel vasculitis.

CT angiography demonstrated a type B aortic dissection, extending from the left subclavian artery to the right superior femoral artery (Figures 1(a) and 1(c)). Visceral perfusion and renal perfusion were adequate. The aorta was dilated up to 4.5 cm at the level of the diaphragm. The right subclavian, left coronary, bronchial, splenic, and celiac arteries were elongated and dilated. The CT scan showed signs of pulmonary emphysema. The anti-MPO ANCA titre remained negative. His renal function was stable and his urine analysis was normal.

In view of multiple enlarged arteries and his medical history that included GCA and glomerulonephritis, an 18-fludeoxyglucose PET-CT scan was performed, which revealed diffuse uptake in the outer layers of all large vessels (Figure 1(e)). Large vessel vasculitis was diagnosed.

## 4. Differential Diagnosis

Differential diagnosis included the following:

- Microscopic polyangiitis
- Polyarteritis nodosa
- Giant cell arteritis
- Takayasu arteritis
- Alpha-1 antitrypsin deficiency

**Figure 2:**
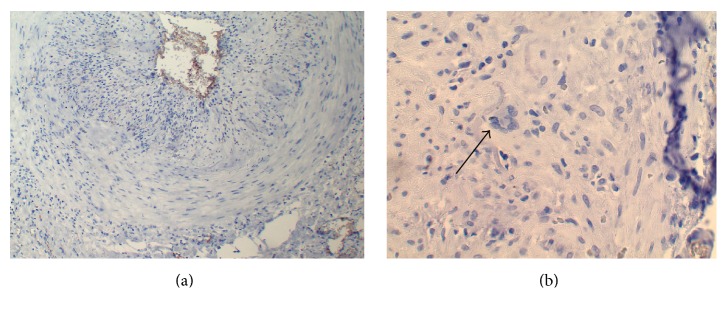
(a) Staining for AAT on temporal artery biopsy. (b) Temporal artery biopsy demonstrating GCA; arrow: giant cell.

AAT was deficient in two separate measurements, both at 0.2 g/L (reference range: 0.9–2.0 g/L) with the ZZ-phenotype determined by electrophoresis. Specimens from the temporal artery biopsy four years earlier were stained using a polyclonal AAT antibody (Dako, Santa Clara, USA) (Figure 2(a)). Two negative controls were used, both from patients diagnosed with temporal GCA but without known AAT deficiency. Compared to controls, in the patient, intense staining of AAT was observed in the endothelium. The findings in the temporal artery biopsy were compatible with the diagnosis of GCA, with observed inflammatory cells in all wall layers and focal infiltration of multinucleated giant cells (Figure 2(b)).

## 5. Treatment and Outcome

The patient was treated conservatively for uncomplicated type B dissection by blood pressure reduction. After the PET-CT scan, 1000 mg of methylprednisolone was administered for three days, followed by prednisolone 60 mg q.d. Follow-up CT scanning of the aorta after 35 days showed no early false lumen dilatation. After one month, pain recurred and further dilatation of the aorta to the left posterior side was seen with mild stranding in the periaortic tissues.

Complex elective endovascular treatment of aortic pathology in patients with suspected connective tissue disorders is controversial because of high reintervention and complication rates [6]. Together with the patient, we decided that the therapy-related morbidity of open aortic repair outweighed the risk of endovascular treatment failure. Thoracic endovascular aortic repair (TEVAR) was performed to cover the 10 mm entry tear to reduce false lumen perfusion. Overlapping stent grafts (Medtronic) were placed from the level of the left subclavian artery extending to the origin of the celiac trunk (Figures 1(b) and 1(d)). Despite initial success, pain recurred and six weeks after the initial procedure the false lumen was embolized. The postoperative recovery was uneventful. The prednisolone dose was tapered to 20 mg daily after a repeated PET-CT scan revealed no residual signs of vasculitis.

Two months later, pain recurred and a CT scan revealed further dilatation of both the native aortic arch and the aneurysm at thoracic and abdominal levels up to 72 mm. As no flow was demonstrated in the false lumen, an endoleak was considered unlikely. Further endovascular treatment with a custom made fenestrated or branched device was considered, but because of the rapid dilatation following previous stenting no therapeutic effect was expected. Open aortic repair was again considered, but, together with the patient, a conservative approach was preferred.

Subsequently, the condition of the patient deteriorated. No further diagnostics were performed; the patient died four months after the initial TEVAR procedure.

## 6. Discussion

Involvement of large vessels is uncommon in AAV, while extracranial GCA has been commonly observed in patients with cranial GCA [7]. In this patient with a type B aortic dissection and recent MPO-AAV, a more generalized arterial disease was suspected when a CT scan showed multiple dilated and elongated arteries. The emphysema and positive family history of emphysema suggested AAT deficiency, which was confirmed.

The paradoxically increased AAT staining at the endothelium is compatible with a deficiency. Polymerized AAT staining has previously been demonstrated in AAT deficient individuals. Polymerization abolishes antiproteolytic activity and might exacerbate the deficiency [8].

In a recent meta-analysis, the Z-allele of the SERPINA1 gene was significantly associated with both PR3 and MPO-AAV [9]. A proposed mechanism through which AAT deficiency exacerbates vasculitis is the priming of neutrophils for activation by ANCA. Neutrophils primed with AAT polymers express increased superoxide production upon stimulation by ANCA [10].

AAT augmentation therapy has not been evaluated in systemic vasculitis secondary to AAT deficiency. As proteolysis in AAT deficiency and neutrophil priming are ongoing processes, continuation of corticosteroid therapy might be beneficial. More research is required to determine the most appropriate therapy in these rare cases.

## Additional Points

Alpha-1 antitrypsin deficiency should be considered in cases of AAV and COPD in nonsmokers.
